# Geographic Disparities in Cancer Incidence in the US Population Aged 20 to 49 Years, 2016–2020

**DOI:** 10.5888/pcd21.230335

**Published:** 2024-05-09

**Authors:** Tesla D. DuBois, Kevin A. Henry, Scott D. Siegel, Shannon M. Lynch

**Affiliations:** 1 Fox Chase Cancer Center, Division of Cancer Prevention and Control, Philadelphia, Pennsylvania; 2 Temple University, Geography and Urban Studies, Philadelphia, Pennsylvania; 3 Christiana Care Health System, Helen F. Graham Cancer Institute, Wilmington, Delaware; 4 Temple University School of Medicine, Center for Biostatistics and Epidemiology, Philadelphia, Pennsylvania

**Figure:**
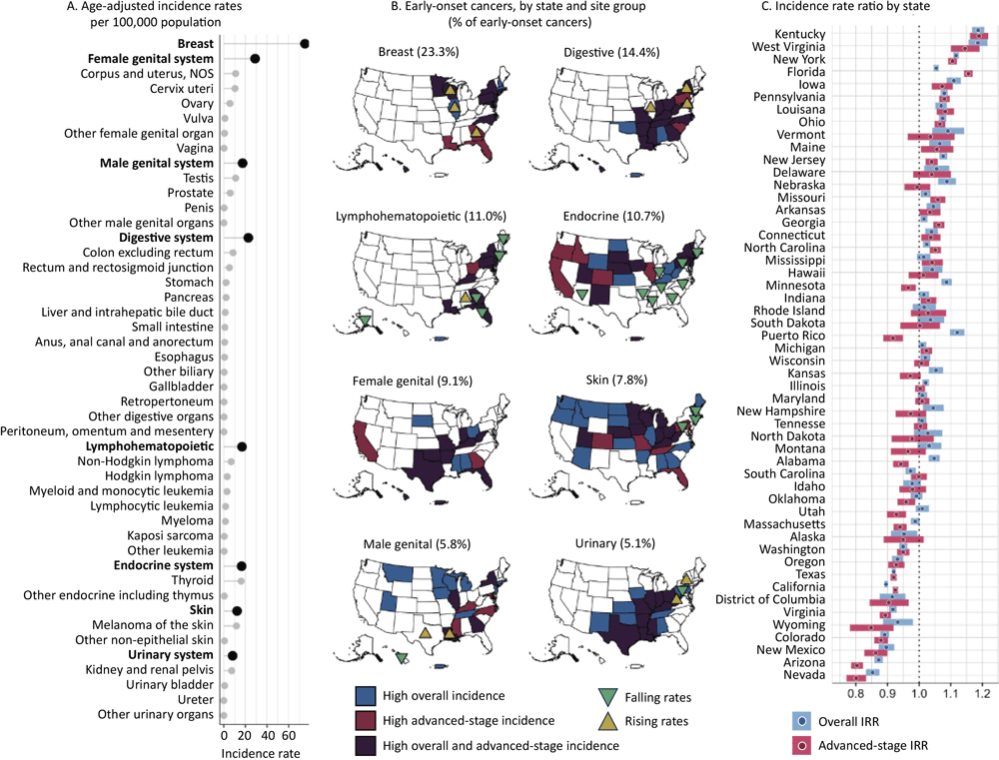
Geographic disparities in cancer incidence in the US population aged 20 to 49 years, 2016–2020. The most prevalent cancer site groups diagnosed among adults aged <50 years are female breast, female genital, male genital, digestive, lymphohematopoietic, endocrine, skin, and urinary. The incidence of early-onset cancers is not distributed evenly across the US. Differing geographic patterns emerge by cancer site group as measured by overall incidence rates, advanced-stage incidence rates, and recent temporal trends. Some states have significantly higher rates of early-onset cancer than the nation overall. In A, dark circles indicate a group of cancer sites; light circles indicate cancer sites within the group. The category Skin excludes basal cell and squamous cell carcinomas. In B, for the male genital group, data on cancer stage were not available for cancer of the testis. In C, shaded bars indicate 95% CIs, and the vertical dashed line indicates the reference group, the US, excluding Puerto Rico. Abbreviations: IRR, incident rate ratio; NOS, not otherwise specified. Data source: Centers for Disease Control and Prevention (1).

**Table:** Figure A is a lollipop chart displaying age-adjusted incidence rates (per 100,000 population) for 8 site groups.

Site	Rate
**Breast**	75.1
**Female genital system**	29.0
Corpus and uterus, NOS	11.0
Cervix uteri	10.4
Ovary	5.7
Vulva	1.1
Other female genital organs	0.7
Vagina	0.2
**Male genital system**	17.4
Testis	11.0
Prostate	6.0
Penis	0.3
Other male genital organs	0.1
**Digestive system**	22.7
Colon excluding rectum	8.6
Rectum and rectosigmoid junction	5.2
Stomach	2.1
Pancreas	2.1
Liver and intrahepatic bile duct	1.3
Small intestine	1.0
Anus, anal canal, and anorectum	0.8
Esophagus	0.7
Other biliary	0.3
Gallbladder	0.2
Retroperitoneum	0.2
Other digestive organs	0.2
Peritoneum, omentum, and mesentery	0.1
**Lymphohematopoietic**	16.9
Non-Hodgkin lymphoma	6.8
Hodgkin lymphoma	3.2
Myeloid and monocytic leukemia	3.0
Lymphocytic leukemia	1.7
Myeloma	1.5
Kaposi sarcoma	0.4
Other leukemia	0.3
**Endocrine system**	16.5
Thyroid	16.1
Other endocrine including thymus	0.5
**Skin excluding basal and squamous**	12.3
Melanoma of the skin	11.7
Other nonepithelial skin	0.6
**Urinary system**	8.2
Kidney and renal pelvis	7.3
Urinary bladder	0.8
Ureter	0
Other urinary organs	0

**Table:** Figure B consists of a series of 8 maps that illustrate which states have higher incidence and/or advanced-stage incidence of cancer compared with the US overall. Each map corresponds to 1 of the 8 site groups.

Cancer site	Rate	Rising	Falling	Compared with US		
				High overall	High advanced-stage	Both high overall and advanced-stage
Breast	23.3	Georgia, Illinois, Wisconsin	None	Delaware, Illinois, Massachusetts, New Hampshire, Rhode Island	Florida, Georgia, Louisiana	Connecticut, Hawaii, Maryland, Minnesota, New Jersey, New York, North Carolina, Pennsylvania, Wisconsin
Digestive	14.4	Illinois, Maryland, New York	None	Alabama, Oklahoma, Puerto Rico	Pennsylvania, South Carolina	Arkansas, Georgia, Hawaii, Indiana, Kentucky, Louisiana, Mississippi, Missouri, New York, North Carolina, Ohio, Tennessee, West Virginia
Lymphohematopoietic	11.0	Alabama	Alaska, Connecticut, Florida, Georgia, Maine	Puerto Rico	Ohio	Connecticut, Florida, Georgia, Kentucky, Louisiana, Maryland, New Jersey, New York, Pennsylvania
Endocrine	10.7	None	Arizona, Arkansas, Georgia, Indiana, Massachusetts, Mississippi, North Carolina, Pennsylvania, Tennessee	Delaware, Kansas, Kentucky, North Dakota, Ohio, Rhode Island, West Virginia, Wyoming	California, Colorado, Idaho, Illinois, Oregon	Connecticut, Iowa, Massachusetts, Nebraska, New Jersey, New Mexico, New York, Pennsylvania, Puerto Rico, South Dakota, Utah, Vermont
Female genital	9.1	None	None	Alabama, Mississippi, Ohio, South Dakota	California, Georgia	Arkansas, Florida, Hawaii, Indiana, Kentucky, Missouri, Oklahoma, Puerto Rico, Texas, West Virginia
Skin	7.8	None	Connecticut, Pennsylvania, New Hampshire	Alabama, Arizona, Arkansas, Georgia, Idaho, Illinois, Kansas, Maine, Montana, North Carolina, North Dakota, Oregon, South Dakota, Washington	Colorado, Delaware, Florida, Missouri, Pennsylvania, Tennessee	Indiana, Iowa, Kentucky, Michigan, Minnesota, Nebraska, New Hampshire, Ohio, Utah, Wisconsin
Male genital	5.8	Louisiana, Texas	Hawaii	Connecticut, Iowa, Michigan, Minnesota, Montana, New Jersey, Puerto Rico, Tennessee, Utah, Wisconsin	Kentucky, Maryland, Mississippi, North Carolina	Georgia, Illinois, Louisiana, New York
Urinary	5.1	New York, West Virginia	Pennsylvania	Alabama, Iowa, Kansas, New Mexico, Oklahoma, Pennsylvania, Tennessee	None	Arkansas, Illinois, Indiana, Kentucky, Louisiana, Mississippi, Missouri, Ohio, Texas, West Virginia

**Table:** Figure C is a stratified forest plot demonstrating how each state compares to the US regarding overall incidence and advanced-stage incidence for all early-onset cancers. States whose 95% CIs do not cross 1 are considered significantly different from the overall US in cancer incidence.

Order	State	Overall incidence rate ratio (95% CI)	Advanced-stage incidence rate ratio (95% CI)
1	Kentucky	1.19 (1.17–1.21)	1.19 (1.16–1.22)
2	West Virginia	1.19 (1.16–1.22)	1.14 (1.10–1.19)
3	New York	1.12 (1.11–1.13)	1.11 (1.09–1.12)
4	Florida	1.05 (1.05–1.06)	1.16 (1.14–1.17)
5	Iowa	1.11 (1.09–1.13)	1.07 (1.04–1.11)
6	Pennsylvania	1.08 (1.07–1.09)	1.08 (1.03–1.10)
7	Louisiana	1.07 (1.05–1.09)	1.08 (1.06–1.11)
8	Ohio	1.07 (1.06–1.09)	1.07 (1.05–1.08)
9	Vermont	1.09 (1.04–1.14)	1.04 (0.96–1.11)
10	Maine	1.06 (1.03–1.10)	1.06 (1.01–1.11)
11	New Jersey	1.08 (1.06–1.09)	1.04 (1.02–1.06)
12	Delaware	1.06 (1.02–1.10)	1.04 (0.98–1.10)
13	Missouri	1.02 (1.01–1.04)	1.06 (1.04–1.08)
14	Nebraska	1.09 (1.06–1.12)	0.99 (0.95–1.04)
15	Arkansas	1.04 (1.02–1.07)	1.03 (1.00–1.07)
16	Georgia	1.02 (1.00–1.03)	1.06 (1.04–1.08)
17	Connecticut	1.04 (1.02–1.06)	1.04 (1.01–1.07)
18	North Carolina	1.02 (1.01–1.04)	1.05 (1.03–1.07)
19	Mississippi	1.02 (0.99–1.03)	1.04 (1.01–1.08)
20	Hawaii	1.04 (1.01–1.07)	1.01 (0.97–1.06)
21	Minnesota	1.09 (1.07–1.10)	0.97 (0.94–0.99)
22	Rhode Island	1.02 (0.98–1.05)	1.03 (0.97–1.09)
23	Indiana	1.02 (1.0–1.03)	1.03 (1.01–1.05)
24	South Dakota	1.04 (1.00–1.0)	1.00 (0.94–1.07)
25	Michigan	1.01 (1.00–1.02)	1.02 (1.00–1.04)
26	Wisconsin	1.02 (1.00–1.04)	1.01 (0.98–1.03)
27	Puerto Rico	1.12 (1.10–1.14)	0.92 (0.89–0.95)
28	Kansas	1.05 (1.03–1.08)	0.97 (0.94–1.00)
29	Illinois	1.02 (1.01–1.03)	1.00 (0.99–1.02)
30	Maryland	1.01 (1.00–1.03)	1.01 (1.00–1.03)
31	New Hampshire	1.04 (1.01–1.08)	0.97 (0.93–1.02)
32	Tennessee	1.01 (1.00–1.02)	1.00 (0.98–1.03)
33	North Dakota	1.03 (0.98–1.07)	0.98 (0.91–1.05)
34	Montana	1.03 (1.00–1.07)	0.96 (0.91–1.02)
35	Alabama	1.05 (1.03–1.06)	0.94 (0.92–0.97)
36	South Carolina	0.97 (0.96–0.99)	1.00 (0.97–1.02)
37	Idaho	0.98 (0.95–1.01)	0.98 (0.94–1.02)
38	Oklahoma	0.99 (0.97–1.01)	0.96 (0.93–0.99)
39	Utah	1.01 (0.99–1.03)	0.93 (0.90–0.96)
40	Massachusetts	0.99 (0.97–1.00)	0.94 (0.92–0.96)
41	Alaska	0.95 (0.91–0.99)	0.95 (0.89–1.02)
42	Washington	0.95 (0.94–0.96)	0.95 (0.93–0.97)
43	Oregon	0.93 (0.92–0.95)	0.93 (0.90–0.95)
44	Texas	0.92 (0.91–0.93)	0.92 (0.91–0.93)
45	California	0.90 (0.89–0.90)	0.92 (0.92–0.93)
46	District of Columbia	0.92 (0.88–0.96)	0.90 (0.84–0.97)
47	Virginia	0.92 (0.90–0.93)	0.89 (0.88–0.91)
48	Wyoming	0.93 (0.89–0.98)	0.85 (0.78–0.92)
49	Colorado	0.89 (0.88–0.90)	0.88 (0.86–0.90)
50	New Mexico	0.90 (0.87–0.92)	0.86 (0.83–0.90)
51	Arizona	0.87 (0.86–0.88)	0.80 (0.78–0.82)
52	Nevada	0.85 (0.83–0.88)	0.80 (0.77–0.83)

## Background

A growing awareness of the increase in the incidence of early-onset cancer, defined as cancer diagnosed in adults aged 50 years or younger (2), has prompted researchers to investigate the underlying drivers of this trend (3). These investigations have focused on racial and ethnic disparities (4) and colorectal (5–8) and breast cancers (9,10). The objective of our analysis was to describe the geospatial patterns of states with a high incidence of early-onset cancer. By identifying priority states and cancer types, our analysis can generate hypotheses about drivers of early-onset cancer and guide prevention and screening interventions.

## Data and Methods

Data for this analysis are from the US Cancer Statistics Public Use Research Database, provided by the National Program of Cancer Registries and the Surveillance, Epidemiology, and End Results programs (1). The analysis included adults aged 20 to 49 with invasive cancer (excluding in situ cases) diagnosed during the 6-year period from 2015 through 2020. We calculated age-adjusted incidence rates (IRs) per 100,000 population for each cancer site by using the 2000 US standard population and, separately, for each cancer site for each state for the 5-year period from 2016 through 2020. Breast cancer and female genital cancer rates were based on the female population, and male genital cancer rates were based on the male population. We calculated incidence rate ratios (IRRs) and associated 95% CIs for the same period for each state by using the national rate as the reference. We calculated a second set of IRs, IRRs, and 95% CIs for advanced-stage early-onset cancer cases diagnosed at regional and distant stages, demonstrating how each state compares to the US in overall incidence and advanced-stage incidence for all early-onset cancers; we considered states whose 95% CIs did not cross 1 to be significantly different from the US rate. We calculated trends as the average annual percentage change (APC) in the 5-year period before the COVID-19 pandemic (2015–2019) to avoid the effect of postponed diagnoses. Trends were significant when the 95% CI for the APC did not cross zero. Negative APCs indicate falling rates, and positive APCs indicate rising rates. The percentage of early-onset cancer cases contributed by each site group and all visualizations were produced in R Statistical Computing Language version 4.3.1. All other analyses were conducted in SEER*Stat version 8.4.2 (R Core Team).

## Highlights

In our study, early-onset cancer (IR = 158.2) accounted for 11.4% of all cancer cases (IR = 599.9), including 17.3% of female breast cancers (overall IR = 177.9) and 8.8% of digestive cancers (overall IR = 108.7). We found that 87.2% of early-onset cancer cases fell into 8 groups of early-onset cancer sites (Panels A and B). Breast cancer contributed 23.3% of all early-onset cancer. Digestive cancers, including colon and rectum sites, contributed 14.4%. Lymphohematopoietic cancers (or “blood cancers”), which include lymphomas and leukemias, contributed 11.0%. Endocrine cancers, predominately thyroid cancer, contributed 10.7%. Female genital cancers, including uterus and cervix sites, contributed 9.1%, and skin (excluding basal and squamous) cancers, predominately melanoma, contributed 7.8%. Male genital cancers, including testis and prostate, contributed 5.8%, and urinary cancers, including kidney and renal pelvis, contributed 5.1%. Three prevalent early-onset cancer sites were not represented in the 8 site groups: lung and bronchus (IR = 4.7), brain (IR = 3.5), and tongue (IR = 1.3).

Our maps of high overall incidence and high advanced-stage incidence indicate that the incidence of early-onset cancer is not evenly distributed (Panel B). States that have worse-than-national rates are frequently near each other geographically. States with changing rates only sometimes have the highest incidence.

The rate of early-onset female breast cancer (IR = 75.1) was worse than the national rate in 17 states, which, except for Hawaii, are located in the eastern half of the US, and rates were rising in 3 states (Georgia, Illinois, Wisconsin) (Panels A and B). Eighteen states had worse-than-national rates of digestive cancers (IR = 22.7). Aside from Hawaii and Puerto Rico, these states are located in the eastern half of the US, with a concentration in the South. Rates of digestive cancers were rising in 3 states (Illinois, Maryland, New York). The incidence of lymphohematopoietic cancers (IR = 16.9) was highest in 3 states in the Southeast, 7 states in the Northeast, and Puerto Rico. Rates were rising in 1 state (Alabama) and falling in 5 (Alaska, Connecticut, Florida, Georgia, Maine). Rates of endocrine cancers (IR = 16.5) were worse than national rates in 25 states, which form a horizontal core of the country running from east to west, plus Puerto Rico. Rates of endocrine cancers were falling in 9 states (Arizona, Arkansas, Georgia, Indiana, Massachusetts, Mississippi, North Carolina, Pennsylvania, Tennessee) and not rising in any. Rates of female genital cancers (IR = 14.5) were worse than national rates in 16 states, largely in the Midwest and South, plus California and Puerto Rico; rates were not rising or falling in any state. Rates of skin cancer (IR = 12.3) were worse than national rates in 32 states, concentrated in the northern portion of the country. Three states had falling rates of skin cancer (Connecticut, New Hampshire, Pennsylvania), and none had rising rates. Rates of male genital cancers (IR = 8.7) were worse than national rates in 18 states, mostly in the eastern half of the country, plus Montana, Nebraska, and Puerto Rico. These rates were rising in 2 states (Louisiana, Texas) and falling in one (Hawaii). Rates of urinary system cancers (IR = 8.2) were worse than national rates in 17 contiguous states, from New Mexico to Pennsylvania. Rates were rising in 2 states (New York, West Virginia) and falling in one (Pennsylvania).

The states with the highest overall and advanced-stage incidence rates of early-onset cancer for all cancer sites combined were Kentucky and West Virginia (Panel C), followed by 13 others that also had worse-than-national rates on both (Arkansas, Connecticut, Florida, Georgia, Iowa, Louisiana, Maine, Missouri, New Jersey, New York, North Carolina, Ohio, and Pennsylvania).

## Action

This study provides the first analysis of age-adjusted rates of early-onset cancer based on state-level population and case counts. Geographic patterns in early-onset cancer indicate possible similarities that could relate to demographic, socioeconomic, behavioral, or environmental risks. By uncovering geospatial patterns across various cancer sites, this analysis informs hypotheses about factors driving early-onset cancer. Because important local patterns may be masked in a state-level analysis, future analyses may consider a more granular geographic unit such as county or zip code. However, focusing prevention efforts on the highest-incidence states for the most prevalent sites may reduce the rate of early-onset cancer nationally.
